# Mediterranean Diet as a Management Strategy for Gestational Diabetes Mellitus: A Narrative Review of the Literature

**DOI:** 10.7759/cureus.98254

**Published:** 2025-12-01

**Authors:** Ammara Kashif, Monica Chekera, Anastasios Malakasis, Vaia Sarli, Nikolaos Machairiotis

**Affiliations:** 1 Department of Obstetrics and Gynecology, Oxford University Hospitals NHS Foundation Trust, Oxford, GBR; 2 Department of Obstetrics and Gynecology, Women’s Centre, John Radcliffe Hospital, Oxford University Hospitals NHS Foundation Trust, Oxford, GBR; 3 Third Department of Obstetrics and Gynecology, Attiko University Hospital, National and Kapodistrian University of Athens School of Medicine, Athens, GRC

**Keywords:** gestational diabetes mellitus, insulin resistance, mediterranean diet, nutrition, pregnancy

## Abstract

Gestational diabetes mellitus (GDM) is a medical complication of glucose intolerance that develops during pregnancy and is among the most common pregnancy complications. If not adequately managed, it can lead to serious fetomaternal complications. Risk factors for developing GDM include maternal age, family history of diabetes, obesity, and diet. Modifiable risk factors, particularly diet, have been extensively studied. Several studies suggest that adherence to the Mediterranean diet (MedDiet) may be associated with a lower incidence of GDM and improved glucose tolerance. This review aims to provide a comprehensive overview of current knowledge on the use of the MedDiet during pregnancy and its potential as a nutritional intervention for managing GDM.

## Introduction and background

Gestational diabetes mellitus (GDM) is characterized as disordered glucose tolerance that develops during pregnancy [1]. In the UK, it is estimated to affect approximately one in 23 pregnancies [2], while global prevalence is reported to range between 7% and 15%, depending on the diagnostic criteria and population studied [1,3,4]. Although the pathophysiology of GDM is not fully understood, it is widely accepted that the fetoplacental unit and pregnancy-induced insulin resistance are the primary driving factors [1,3]. GDM is associated with adverse pregnancy outcomes and increased long-term morbidity for both mother and child, including macrosomia, preeclampsia, cesarean delivery, and an elevated lifetime risk of type 2 diabetes mellitus and metabolic syndrome [1,3,5,6]. Non-modifiable risk factors for GDM include maternal age, previous GDM, and family history of type 2 diabetes mellitus [1,4]. Identifying modifiable risk factors is crucial for implementing preventive strategies to reduce the risk of developing GDM and its associated complications. One such modifiable risk factor is diet.

GDM represents a state of glucose intolerance that develops or is first recognized during pregnancy [1]. Its prevalence is increasing globally, paralleling trends in obesity and sedentary lifestyles [1,3,6]. Although the condition typically resolves after delivery, it carries significant short- and long-term implications for both mother and child, including increased perinatal morbidity, higher rates of operative delivery, and a markedly increased risk of future type 2 diabetes mellitus in the mother [1,5,6]. Offspring of pregnancies complicated by GDM are also at greater risk of obesity, impaired glucose tolerance, and cardiometabolic disease later in life [5,6]. These observations underscore the importance of early identification and effective management of GDM, as well as preventive strategies targeting high-risk women.

The pathophysiology of GDM involves pregnancy-induced insulin resistance that surpasses the adaptive threshold of maternal β-cell function [1,3]. Placental hormones, such as human placental lactogen, progesterone, estrogen, and cortisol, play a central role by antagonizing insulin action, while maternal factors such as preexisting obesity, chronic low-grade inflammation, and genetic predisposition further amplify glucose dysregulation [1,3,6]. Consequently, identifying modifiable lifestyle determinants, particularly diet and physical activity, has become crucial in mitigating the risk of GDM and its sequelae [1,6-8].

Among modifiable factors, diet is particularly influential. Ancel Keys first defined the Mediterranean diet (MedDiet) as a dietary pattern low in saturated fat and high in vegetable oils [9]. The MedDiet is characterized by a high intake of fruits, vegetables, whole grains, legumes, nuts, and extra-virgin olive oil (EVOO); moderate consumption of fish and poultry; and limited intake of red and processed meat and refined sugars [9-11]. This dietary pattern is rich in monounsaturated and polyunsaturated fatty acids, polyphenols, antioxidants, and fiber, which are thought to improve insulin sensitivity, modulate inflammation, and confer cardiovascular and metabolic protection [10-13]. These mechanisms provide biologically plausible pathways through which the MedDiet might prevent or ameliorate GDM.

It is important to treat GDM not only to reduce serious perinatal morbidity but also to improve women’s long-term health and quality of life [5,6]. Treatment strategies for women with GDM include medical nutrition therapy (MNT) and pharmacotherapy [5,7]. Approximately two-thirds of women with GDM achieve targeted glycemic control and favorable pregnancy outcomes through diet modification alone [5,7]. In cases where MNT does not achieve the desired effect, oral hypoglycemics and insulin can be used [5,7]. Although the search for the optimal dietary pattern is ongoing, current evidence suggests that the MedDiet is one of the most promising approaches [8,9].

While it is difficult to standardize dietary interventions due to variable eating habits across regions, the MedDiet has been successfully adapted by researchers to make it acceptable for different study populations, with promising results [9-11,14-16]. Most studies focus on the MedDiet as a preventive strategy for GDM, but extrapolating data from nonpregnant populations and high-risk groups suggests it may also be a reasonable strategy for managing established GDM [7-9,17]. This narrative review will provide an overview of the general health benefits of the MedDiet, its use in pregnancy, fetomaternal benefits, and specifically its role as a treatment modality for GDM, along with barriers to implementation and areas for future research.

## Review

Benefits of the MedDiet in pregnancy

Observational studies from Italy, including 2,000 adults, showed that adherence to the MedDiet was associated with improved quality of life and sleep, primarily through weight reduction [15]. Evidence of the diet’s beneficial effects on the cardiovascular and neurological systems, as well as on diabetes and dyslipidemia, is of mixed quality [16,17]. Additionally, some studies suggest that the MedDiet may reduce the risk of developing and recurrent cancer [18]. Building on these findings, research has extended to examine the fetomaternal effects of this eating pattern before and during pregnancy, with promising results.

Biagi et al. conducted a systematic review investigating the effect of the MedDiet during pregnancy on perinatal outcomes and child health [19]. They concluded that a high-quality maternal diet in general, and adherence to the MedDiet in particular, can reduce the incidence of neural tube defects, fetal growth restriction, small-for-gestational-age (SGA) infants, and prematurity [20]. Evidence regarding protection from childhood asthma and allergies is heterogeneous, but there is growing support for beneficial effects of the MedDiet during pregnancy on children’s health.

The positive impact of the MedDiet on cardiometabolic profiles has prompted researchers to investigate its role in reducing the risk of GDM. Makarem et al. conducted a cohort study in a diverse ethnic population in the US and proposed that adherence to the MedDiet is inversely related to adverse pregnancy outcomes. They concluded that interventional studies are needed to confirm these findings [13].

The St. Carlos GDM Prevention Study, a randomized controlled trial (RCT) conducted by Assaf-Balut et al., used the MedDiet supplemented with EVOO and pistachios as an intervention to reduce the risk of GDM [20]. They observed a statistically significant reduction in the incidence of GDM in the intervention arm, as well as lower rates of insulin-treated GDM, prematurity, gestational weight gain at 24-28 and 36-38 weeks, emergency cesarean sections, perineal trauma, and both SGA and large-for-gestational-age (LGA) newborns.

Another interventional study in China adapted the MedDiet to local dietary habits by replacing olive oil with rapeseed oil and including red meat to address the high prevalence of iron-deficiency anemia in their population [14]. The intervention began at recruitment, and women who conceived within six months were excluded to allow adequate time for the intervention to exert its effects. The results of this RCT were consistent with earlier studies, suggesting a reduced incidence of GDM with high adherence to the MedDiet.

MedDiet in gestational diabetes

GDM is rising globally and is associated with fetomaternal complications such as macrosomia, intrapartum complications, and an increased risk of type 2 diabetes. NICE and ADA guidance recommend first-line management with healthy eating and physical activity, which can be effective for some women in achieving glycemic targets [3,21]. A systematic review and meta-analysis exploring the influence of a low glycemic index (GI) diet on GDM concluded that, compared with control interventions, a low-GI diet significantly reduced postprandial glucose but did not show notable effects on fasting plasma glucose, HbA1c, birth weight, macrosomia, or insulin requirements [22]. A 2017 Cochrane review that included 11 RCTs involving 2,786 pregnant women concluded that there was insufficient evidence to determine whether any single dietary intervention was effective. Since then, there has been increasing focus on the MedDiet in pregnancy, highlighting the need for further review.

The St. Carlos GDM Prevention Study, an RCT involving low-risk Spanish women, concluded that early nutritional intervention with the MedDiet reduces the incidence of GDM and adverse maternal-fetal outcomes, suggesting it should be universally applied as first-line therapy [20]. The Finnish GDM Prevention Study, RADIEL, was another RCT targeting women at high risk for developing GDM [23]. The intervention began early in pregnancy at 13 gestational weeks and included physical activity. While some studies implemented dietary interventions prior to pregnancy [13,14], both the St. Carlos and RADIEL RCTs recruited women at their first prenatal visit. Both trials reported a reduced risk of GDM with the MedDiet; however, in the RADIEL trial, the intervention included both diet and physical activity, so the reduction in GDM incidence cannot be solely attributed to the MedDiet [23,24].

Melero et al. implemented the MedDiet at the first antenatal visit to determine whether it reduces the incidence of GDM and adverse maternal-fetal outcomes in Hispanic women in Spain, with follow-up extending to three years postpartum [25]. The study demonstrated that initiating this nutritional intervention early in pregnancy not only reduces the rate of GDM but also provides metabolic benefits up to three years postpartum compared to interventions commenced after delivery. This approach highlights pregnancy as an opportunity to educate women and promote lasting changes in nutrition and general health practices.

Al Wattar et al. conducted the first multicenter RCT using the MedDiet in pregnancy, enrolling 1,252 inner-city pregnant women from five centers in the UK who had metabolic risk factors [26]. Participants received individualized dietary advice at 18, 20, and 28 weeks’ gestation. The primary endpoints were composite maternal outcomes (including GDM or preeclampsia) and composite fetal outcomes (including stillbirth, SGA, or neonatal intensive care admission). The study found that women following the MedDiet had lower gestational weight gain and reduced risk of GDM, but there was no difference in adverse pregnancy outcomes.

This observation contrasts with the prospective multicenter cohort study by Makarem et al., which recruited 10,038 women and implemented the dietary intervention during the preconception period [13]. They reported that greater adherence to the MedDiet was associated with a lower risk of adverse pregnancy outcomes. In current clinical practice, pre-pregnancy interventions may not always be feasible for high-risk populations. Combining insights from these multicenter studies, it can be inferred that initiating the MedDiet during pregnancy can reduce both gestational weight gain and the risk of GDM.

An observational study by Karamanos et al. concluded that adherence to a MedDiet is associated with a lower incidence of GDM and improved glucose tolerance, even in women without GDM [27]. This study assessed the dietary habits of 1,076 pregnant women across 10 Mediterranean countries using a validated questionnaire. From this, a Mediterranean Diet Index (MDI) was computed to reflect adherence to the MedDiet pattern, with higher scores indicating better adherence. After adjustment for age, BMI, family history of diabetes, weight gain, and energy intake, women with GDM had lower MDI scores. Moreover, the incidence of GDM was lower among women with better adherence to the MedDiet.

Across the reviewed literature, adherence to the MedDiet during pregnancy is consistently associated with improved metabolic and obstetric outcomes, although the magnitude of benefit varies depending on the timing, intensity, and cultural adaptation of the intervention. RCTs, such as the St. Carlos GDM Prevention Study and the RADIEL trial, demonstrated significant reductions in GDM incidence, ranging from 25% to nearly 40%, among women receiving structured MedDiet counseling compared to standard dietary advice. These trials also reported favorable secondary outcomes, including lower gestational weight gain, reduced insulin requirements, and decreased rates of LGA infants.

Observational and cohort studies support these findings, linking higher MedDiet adherence scores with lower fasting glucose, improved lipid profiles, and reduced markers of systemic inflammation. For example, Makarem et al. and Karamanos et al. reported inverse associations between MedDiet adherence and both GDM incidence and glucose intolerance, independent of maternal BMI or baseline metabolic risk [13,27]. Importantly, studies that initiated dietary interventions early in pregnancy, or even preconceptionally, tended to show stronger effects, suggesting a critical window during which dietary modification exerts maximal benefit.

Meta-analytical evidence remains limited due to heterogeneity in study design and adherence indices; however, pooled data indicate a relative risk reduction of approximately 30% in GDM development among women following a MedDiet-like pattern. Additionally, neonatal outcomes, such as lower rates of macrosomia, preterm birth, and neonatal intensive care admissions, have been reported in several trials, though findings are not uniform across all populations. Collectively, these findings highlight the MedDiet as a promising, nonpharmacological intervention that offers both metabolic and obstetric advantages when implemented early and maintained consistently throughout pregnancy. Based on the available studies, it can be concluded that the MedDiet can be beneficial for women at risk of, or with a diagnosis of, GDM; however, it should be initiated early in pregnancy to maximize benefits.

There is currently no consensus regarding the ideal MNT for treating GDM, although the MedDiet appears promising. Yamamoto et al. performed a systematic review and meta-analysis of RCTs examining the impact of modified dietary interventions on maternal glucose control and neonatal birth weight but did not include the MedDiet, likely due to limited data [7]. Their analysis included 18 studies with a total of 1,151 pregnant women with GDM and concluded that low-GI diets were associated with larger decreases in fasting and postprandial glucose compared with standard diets. Regarding fasting and postprandial glucose, gestational weight gain, fetal weight, HOMA-IR, and HbA1c, the DASH diet demonstrated significant benefits. Given the similarities between the DASH diet and the MedDiet, these results can be extrapolated, suggesting that the MedDiet may offer improved glycemic control in GDM.

Women with GDM are also at risk of having SGA infants, potentially due to insufficient weight gain when adhering to restrictive diets. One possible explanation is that substituting energy-dense foods with healthier alternatives may inadvertently reduce caloric intake. Before recommending a specific dietary intervention, more robust evidence is needed; therefore, further research is required to evaluate the effect of the MedDiet as a therapeutic approach for GDM.

Several dietary interventions have been investigated in women with or at risk for GDM, with varying effects on glycemic control and pregnancy outcomes. Among these, the MedDiet has demonstrated consistent benefits compared with other commonly recommended nutritional patterns, such as the low-GI diet, the Dietary Approaches to Stop Hypertension (DASH) diet, and low-carbohydrate regimens.

Low-GI diets have been shown to reduce postprandial glucose excursions; however, evidence regarding their effect on fasting glucose, insulin resistance, and neonatal outcomes remains inconsistent. The DASH diet, which emphasizes fruit, vegetable, and low-fat dairy consumption with sodium restriction, has shown moderate benefits in lowering blood pressure and improving lipid profiles in pregnancy, but limited data support its role in glucose metabolism. Low-carbohydrate diets may achieve short-term reductions in glucose levels; however, excessive carbohydrate restriction during pregnancy can increase ketone production and is therefore not routinely recommended.

Compared with these approaches, the MedDiet provides a balanced macronutrient profile with a favorable fat composition, rich in monounsaturated and polyunsaturated fatty acids, alongside high antioxidant and fiber content. Meta-analyses in nonpregnant populations have demonstrated superior improvements in insulin sensitivity (HOMA-IR), inflammatory markers, and lipid ratios with the MedDiet relative to control diets. The MedDiet also promotes better long-term adherence due to its palatability and flexibility across cultural contexts, which is critical for sustaining glycemic control throughout pregnancy. Consequently, while several dietary models offer partial metabolic benefits, the MedDiet appears to provide the most comprehensive cardiometabolic protection and remains the most evidence-based dietary framework for the prevention and management of GDM.

Challenges for the implementation of the MedDiet in pregnancy

Researchers have encountered challenges in implementing the MedDiet early in pregnancy, often due to women missing their initial prenatal appointments [26]. Dietary habits vary widely with ethnicity, culture, region, and religious beliefs. Additional influences include family pressure, advice from peers or friends, and information from social media forums. Food preferences and allergies can also shape dietary choices during pregnancy. Cost is another important factor, as EVOO is substantially more expensive than ordinary cooking oils, potentially affecting adherence. Some interventional studies addressed this by providing women with EVOO for the entire family and sachets of nuts [28]. Future research on MedDiet in pregnancy should consider the feasibility and cost of providing such support.

Although recent evidence positions the MedDiet as one of the most promising nutritional interventions for the prevention and management of GDM, several knowledge gaps remain. Future studies should prioritize large, multicenter RCTs with standardized definitions of MedDiet adherence to improve comparability. Incorporating objective biomarkers of dietary intake, such as plasma carotenoids, fatty acid profiles, and polyphenol metabolites, could enhance dietary assessment accuracy and strengthen causal inference.

Long-term follow-up studies are also needed to determine whether adherence to the MedDiet during pregnancy translates into sustained maternal metabolic benefits, including reduced incidence of type 2 diabetes mellitus, cardiovascular disease, and metabolic syndrome. Similarly, investigating transgenerational effects on offspring adiposity, insulin sensitivity, and epigenetic regulation would provide valuable insight into early-life metabolic programming.

From a clinical perspective, integrating structured MedDiet counseling into routine antenatal care may offer a cost-effective and culturally adaptable strategy for GDM prevention. The diet’s emphasis on whole foods and plant-based fats aligns with existing public health recommendations and can be tailored to local culinary traditions. Collaboration between obstetricians, dietitians, and primary care providers is essential for effective implementation and monitoring. Ultimately, defining evidence-based nutritional protocols could bridge the gap between lifestyle counseling and pharmacologic therapy, establishing the MedDiet as a cornerstone of metabolic health during pregnancy (Table 1).

**Table 1:** Benefits and challenges of MedDiet MedDiet, Mediterranean diet

Benefits	Challenges
Improves glycemic control	Issues with implementation
Weight reduction	Varying dietary habits
Protective against cardiovascular disease	Cost
Reduces risk of dementia	Societal pressure
Reduces risk of certain types of cancer	

Pathophysiology of GDM

The metabolic changes of pregnancy naturally increase insulin resistance, ensuring a continuous supply of glucose to the fetus. In most women, the pancreas adapts by producing more insulin. In GDM, this balance does not hold: insulin resistance rises beyond the expected level, while β-cell compensation becomes insufficient, leading to maternal hyperglycemia.

Several elements contribute to this shift: placental hormones, low-grade inflammation, altered adipokines, and increased metabolic demands such as enhanced amino acid turnover. Women with underlying risk factors (age, genetics, polycystic ovary syndrome, family history, and lifestyle) start pregnancy with reduced metabolic reserve, making them more vulnerable to developing GDM as normal physiologic changes progress.

**Figure 1:**
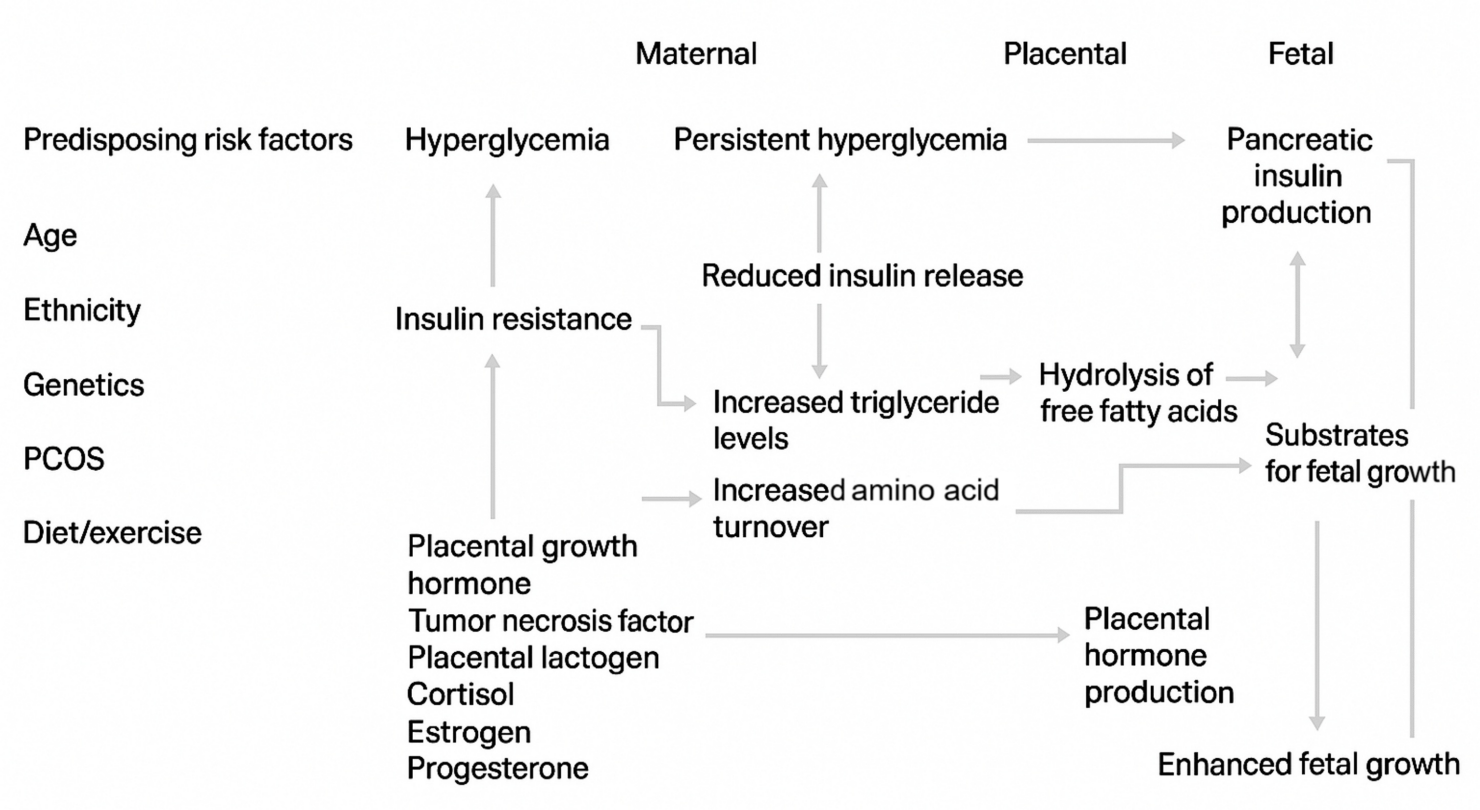
Pathophysiology of GDM This schematic diagram illustrates the interplay between maternal, placental, and fetal factors contributing to insulin resistance and hyperglycemia during pregnancy. GDM, gestational diabetes mellitus; PCOS, polycystic ovary syndrome

Figure 1 summarizes how these maternal, placental, and fetal factors interact. 

## Conclusions

GDM remains a significant global health challenge, contributing to both short- and long-term maternal and neonatal morbidity. Evidence from randomized and observational studies consistently supports the MedDiet as a safe, feasible, and metabolically beneficial dietary model for women at risk of, or diagnosed with, GDM. Early adoption of the MedDiet during pregnancy appears to improve glycemic control, limit excessive gestational weight gain, and reduce the need for pharmacologic interventions in many cases. Beyond pregnancy, adherence to this dietary pattern may provide sustained metabolic benefits, including a lower risk of type 2 diabetes and cardiovascular disease, while promoting healthy lifestyle habits within the family environment. However, heterogeneity across studies, with respect to MedDiet definitions, adherence scoring, and population characteristics, limits the ability to derive universal recommendations. Future research should aim to harmonize study protocols, include diverse populations, evaluate long-term maternal and offspring outcomes, and incorporate objective biomarkers of dietary intake. In the meantime, the available evidence justifies recommending a Mediterranean-style dietary pattern as an integral component of lifestyle counseling for women with, or at high risk of, GDM.
